# Filamentous Bacteriophage—A Powerful Carrier for Glioma Therapy

**DOI:** 10.3389/fimmu.2021.729336

**Published:** 2021-09-10

**Authors:** Yicun Wang, Jiyao Sheng, Jin Chai, Cuilin Zhu, Xin Li, Wei Yang, Ranji Cui, Tongtong Ge

**Affiliations:** Jilin Provincial Key Laboratory on Molecular and Chemical Genetic, The Second Hospital of Jilin University, Changchun, China

**Keywords:** filamentous bacteriophage, glioma, target peptide, antibody, BBB, BBTB

## Abstract

Glioma is a life-threatening malignant tumor. Resistance to traditional treatments and tumor recurrence present major challenges in treating and managing this disease, consequently, new therapeutic strategies must be developed. Crossing the blood-brain barrier (BBB) is another challenge for most drug vectors and therapy medications. Filamentous bacteriophage can enter the brain across the BBB. Compared to traditional drug vectors, phage-based drugs offer thermodynamic stability, biocompatibility, homogeneity, high carrying capacity, self-assembly, scalability, and low toxicity. Tumor-targeting peptides from phage library and phages displaying targeting peptides are ideal drug delivery agents. This review summarized recent studies on phage-based glioma therapy and shed light on the developing therapeutics phage in the personalized treatment of glioma.

## Introduction

Glioma is the most common cerebral malignancy with high morbidity and mortality. Despite the current treatment measures such as surgery, radiation, and chemotherapy, the prognosis and mortality of patients has not improved significantly (1–3). The annual death rate in China is as high as 30,000. Currently, the adverse reactions to glioma drugs are more prominent, and drug resistance is readily developed (1, 4, 5). To overcome the limits of existing therapies, there is a pressing need for a treatment strategy that can selectively target cancer tissues and avoid non-target tissues.

In addition, the blood-brain barrier (BBB) is a formidable obstacle for the transport of most administered therapeutics to the brain (6, 7), and most anti-tumor drugs have difficulty passing the BBB and the blood-brain tumor barrier (BBTB), it is a major hurdle in the development of targeted drugs for glioma (8–10). Therefore, choosing a carrier that can pass through the BBB is very important for glioma treatment.

Filamentous bacteriophages (Ff phage) are nano-scale viruses that infect bacteria and are not harmful to humans (11–13). Ff phage fd, M13, and f1 are stable under harsh conditions and can be manufactured with uniform specifications and low cost (14–17). As well, Ff phage has genetic flexibility. In 1985, Smith et al. reported phage display technology to display a variety of proteins, antibodies, and peptides on the phage coat proteins. Subsequently, phage display libraries were injected intravenously into laboratory animals to screen the targeting peptides (18). Moreover, Ff phage could enter the central nervous system (CNS) without visible toxic effects (19, 20), it can pass through the BBB as a drug carrier, when administered intranasally or through convection-enhanced delivery (CED), and has great research potential for the treatment of brain diseases (21–24).

Furthermore, the phage display library is used quickly and directly to screen peptides targeting tumor and anti-tumor antibodies. To date, there are numerous studies by the phage library screening tumor targeting peptides for target therapy and immunotherapy (25–27), and it has established the method for screening glioma targeting peptides across the BBB, guiding the immunotherapy in patients. These phages and peptides targeting glioma cells could avoid or reduce the toxic effects of anti-cancer drugs (28–31). At the same time, phages, carrying targeted peptides and antibodies, stimulate the immune response and play an immunotherapy role (28, 32–35).

In summary, this review will clarify the strategy for applying Ff phage nanoparticles to glioma treatment. It can be used to direct clinical treatment of tumors and provide new ideas for personalized disease therapy.

## Biopanning the Tumor-Targeted Peptide

**Figure 1:**
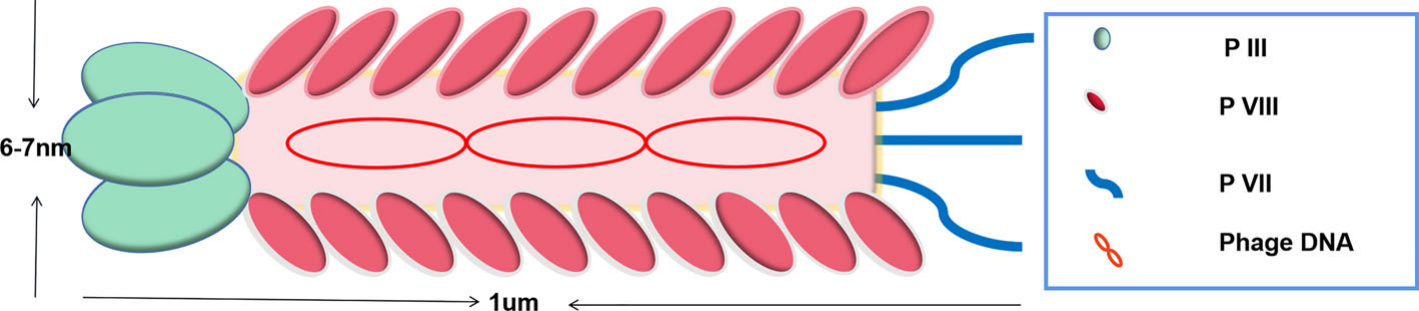
Schematic of Ff phage. Phage consists of a tubular protein coat surrounding a single-stranded circular DNA. Proteins III and VII are the minor coat proteins, present in 3-5 copies. Protein VIII is the major phage coat protein and presents in 2700 copy numbers.

Ff phage is a biological nanomaterial with a length of about 1 um and a diameter of about 7 nm (14, 36). It could specifically infect bacteria and is present in the human body and harmless to humans. Ff phage is made of single-stranded circular DNA and coat proteins. The main coat protein pVIII is located on the phage side and minor coat proteins (pIII, pV I, pVII, and pIX) are located at both tips (**Figure 1**).

Phage display is to insert the DNA sequence of the exogenous peptide into the phage coat protein gene and express the peptide on the surface of the phage along with the expression of the coat protein. The phage displaying peptide still has protein assembly and infection activity (37–39). Based on phage display technology, phage libraries were built and used to select targeting phages, such as tumor-targeted peptides, which improved research efficiency and reduced costs. In addition, these targeted peptides developed functions of cell-targeting, tumor-homing, and cell-penetrating (40–42). Phage libraries were usually screened by using molecules, cells, and tissues *in vitro* or in animals and human patients (38, 43, 44).

Traditional chemotherapeutics have poor accuracy on tumor cells and are prone to adverse reactions. Therefore, the targeted therapy is particularly important for tumor therapy (45–47). Peptides specifically binding to tumor tissues, as carriers to direct drugs to tumor tissues, significantly improved the accuracy of drug targeting (48–51). Although monoclonal antibodies as vectors were successfully applied to anti-tumor, the high molecular weight of antibodies might reduce efficiency (52–55), while the phage peptide library has the benefits of screening for small molecular weight peptides, which can compensate for antibody deficiencies that are widely used in the diagnosis and treatment of glioma.

The screened peptides could combine with markers for imaging. Wang et al. developed an HO-8910 ovarian cancer cell targeting peptide (NPMIRRQ) from the phage library, which demonstrated the ability to selectively bind ovarian cancer cells using immunofluorescence and immunohistochemical assays (56).

The screened peptides could also couple with chemotherapeutic drugs or some gene, and then be used in the tumor-targeted treatment or gene treatment; Du et al. obtained the A54 peptide (AGKGTPSLETTP) by *in vivo* phage display for hepatocarcinoma and conjugated it with doxorubicin for *in vivo* targeted therapy. The study showed the A54-doxorubicin reduced the tumor size and prolongated the long-term survival rate (57).

Furthermore, some specific binding peptides that inhibit tumor growth, invasion, and metastasis, could be used to treat tumors directly. Zhou et al. isolated the peptide, SWQIGGN, from a Ph.D.-C7C phage library with the ovarian cancer cell HO-8910 (58). They found that the peptide controlled cancer cell migration, viability, adhesion capacity, invasion, and tumor growth *in vivo*.

Currently, the screening of tumor-targeted peptides is widely used in targeting the treatment of tumors, such as lung cancer, stomach cancer, liver cancer, colon cancer, and prostate cancer.

## Ff Phage, a Targeted Therapy Vector

Poor permeability of the cellular plasma membrane to a drug or gene is the main barrier for targeted delivery, while the nature of vectors affects the efficiency of drug delivery in tumors and tumor-affected tissues. Therefore, construction and selection of drug vectors is one of the most important steps of tumor therapy. Ff phage could deliver genes and peptides to mammalian cells, and the structure of Ff phage results in more efficient cellular attachment and ensuing membrane penetration. It has been successfully used in treatment under the U.S. Food and Drug Administration (FDA) process (www.fda.gov), and methods for isolating, storing, and producing phages are now becoming more available and better developed under the ATCC (www.atcc.org) and PHE (www.gov.uk/government/organisations/public-health-england) collections.

Ff phage might be an ideal carrier for drug therapy and immunotherapy. First, an exogenous gene could be inserted into the Ff phage genome. Meanwhile, the peptide displayed on the Ff phage presents its natural conformation and the phage has a strong resistance to physical and chemical factors (59–61). Second, the phage displayed exogenous peptides or chemical modifications, which could combine with inorganic nanomaterials/drugs, to form phage-nanocomplexes and drug-loaded phages. It is well utilized in photodynamic cancer therapy (62–64). Some researchers used the Fd phage to display a cancer-targeting peptide on pVIII major coat protein, and then conjugated photosensitizer at the N-terminal end of the targeting peptides, and demonstrated that the complex of phage-photosensitizers was able to selectively target and kill SKBR3 tumor cells *in vitro* (65). Third, the displayed Ff phage triggers every arm of the immune response. Berardinis et al. engineered fd to target mouse dendritic cells (DCs), and activated the innate and adaptive responses without the need of exogenous adjuvants (66). The study has also shown that phage could induce the IL-2 and IFN-γ cytokines, which were useful in tumor immunotherapy (67). Fourth, drug conjugated phage increases the half-life in the blood steam (68), while the toxicity and side effects of hazardous drugs are reduced in combination with the Ff phage.

In a word, Ff phage, as a carrier of therapeutic reagents, has more advantages in targeted therapy, with high specificity, high sensitivity, and reproducibility.

## The Application of Phage Nanomaterials to Glioma Therapy

Gliomas are aggressive brain tumors and challenging therapeutic cancers that have high mortality (69). The 5-year survival rate of glioma is very low (70), and the prognosis of glioblastoma patients is poor with a median survival of less than 1 year. Recently, cancer research in the U.K. showed that 40% of brain tumor patients survive their cancer for 1 year and more than 10% survive their cancer for 5 years or more.

At present, the clinical therapies for gliomas are surgical therapy, radiation therapy, chemotherapy, gene therapy, and other comprehensives (1, 71–74). However, it is easy to relapse after these treatments, and the patients’ survival rates are not significantly improved. Immunotherapy is useful for treating tumors, the mAbs bevacizumab, rituximab, and trastuzumab were already widely used against tumors outside the brain (75–79). But the current immunotherapy for medical glioma is costly and inefficient.

BBB is another significant barrier to the delivery of targeted treatments for brain tumors. Indeed, more than 98% of low-molecular-weight candidate drugs and almost 100% of large therapeutic candidate drugs cannot cross the BBB (80). There is an urgent need for a carrier that carries drugs across the BBB. Phage display technology can be used for the construction of peptide libraries to screen for glioma tumor-targeting peptides and peptides across the BBB. Surface functionalization with these peptides is a sophisticated way to develop drug delivery platforms that cross the BBB and target glioma.

### Identification of Targeting Peptide and Antibody

About 30% of all human antibody therapies are derived from phage antibody libraries. In addition, the screening of phage display libraries is an effective tool to obtain peptides that target glioma tumors both *in vitro* and *in vivo* (43, 49, 81, 82).

#### *In Vitro* Panning

*In vitro* panning was used to identify peptides that specifically bind to glioma cells and proteins. Ho et al. isolated GL1 peptide that specifically interacts with primary glioma cells obtained from human biopsy specimens using a phage library and injected the GL1-bearing phages into a mouse (83). They found that the phage targeted the mouse brain tumor and this peptide had the potential to be used for therapeutics to glioma cells.

Glioma stem cells (GSCs) are the major drivers of brain tumors. Beck et al. screened the peptides binding to GSCs from the phage display library, and the administration of GSC-homing peptide into the glioma mice model resulted in penetration into the brain and specific accumulation in glioma. CD133 is a cell surface antigen allowing identification of GBMs. Yoon et al. screened the peptides targeted CD133 from U373 glioma cells, using the phage library, and conjugated the targeting peptide (CBP4) to GNPs. They found that the targeting peptide was effective for passage into the brain extracellular space (84). The protein kinase C (PKC) family plays an important role in glioma, is a potential biomarker to disturb the expression of CD133 on glioma cells, and may have a therapeutic effect on GSCs. Yoon et al. also identified 12-amino-acid peptide-binding toward the PKCd catalytic domain through a phage display library and certificated that the peptide could target and inhibit PKC, provided a novel peptide sequence for a therapeutic strategy to target GSCs. To identify novel peptides targeting malignant gliomas, Wang et al. used a 12-mer peptide phage display library and obtained the peptide (VTWTPQAWFQWV) bound to U87MG cells. In addition, the VTW phage is bound strongly to other human glioma cell lines, including H4, SW1088, and SW1783 (85–87).

The discovery and isolation of antibodies are important for the treatment of glioblastoma (GBM). Insulin-like growth factor binding protein 2 (IGFBP2) is highly upregulated in GBM tissues and plays a crucial role in the invasion of glioma cells. Kondaiah et al. screened scFv phage display libraries using recombinant IGFBP2 and identified that scFv B7J could bind to IGFBP2 and inhibit the migration and invasion of glioma cells (88). Tumor sphere cells more closely resemble the phenotype of primary tumors than do serum-cultured cell lines. Liu et al. derived GBM tumorspheres from human brain tumor specimens, biopanning the scFvs that bind to CD133 positive GBM tumorsphere cells from scFvs phage library and indicated one scFvs could inhibit the growth of the GBM tumorsphere cells *in vitro*.

Overall, peptides targeting glioma were identified using phage display library *in vitro*. It is useful for further development of novel therapies that target glioma cells and provide novel diagnostic and therapeutic modalities for human brain malignancies.

#### *In Vivo* Pannings

*In vivo* pannings were successful in obtaining organ-specific targeting peptides in the animal model. Peptides and antibodies may be isolated, which recognize subsets of glioma tumors *via in vivo* biopanning of phage display libraries in glioma xenografts.

GBM displays cellular hierarchies with self-renewing glioma-initiating cells (GICs) at the apex. To discover new GIC targets Rich et al. delivered a phage peptide library intravenously to a GBM xenograft *in vivo*, then derived GICs, and then identified the peptides targeting VAV3 and CD97. These peptides could be used for identifying and targeting of GICs (89),

Additional destruction of existing tumor vasculature effectively deprives tumors from blood. With the need to identify novel tumor vascular targeting agents, Lith et al. identified a nanobody C-C7 *in vivo* biopanning of phage display library in an orthotopic mouse model of diffuse glioma, which showed that C-C7 recognized a subpopulation of tumor blood vessels in glioma xenografts and clinical glioma samples (90). Leenders et al. cloned a nanobody phage library from lymphocytes of a llama, which had been immunized with clinical glioma tissue and isolated the nanobodies discriminated incorporated pre-existent vessels in highly infiltrative cerebral E434 xenografts from normal brain vessels *via* biopanning *in vivo* with this library in the orthotopic glioma xenograft models (91).

*In vivo* biopanning, in appropriate animal models, is a very promising approach for future identifying novel molecular tools for targeting glioma tumors and oncogenic pathways preferentially activated within the tumor hierarchy, which could offer a new strategy for the development of glioma therapy.

### Development of Carriers for Targeted Drug Delivery

The BBB and BBTB restrict the entry of drugs given routinely with glioma (92, 93). Thus, effective glioma treatment requires therapeutic agents to penetrate both BBB and BBTB. An emerging solution consists of identifying the peptide vectors that penetrate the BBB/BBTB.

In recent years, numerous studies have focused on modifying the pharmacokinetics of chemotherapeutic drugs by using a delivery vector or by adding targeting properties. Langel et al. developed a tumor-targeted delivery vector gHoPe2 that is based on a cell-penetrating peptide pVEC and a novel glioma-targeting peptide sequence gHo (NHQQQNPHQPPM), which was identified using phage display technology. The vector could be efficiently absorbed into glioma cells and xenograft glioma tumors in a mouse model. In addition, vectored doxorubicin was more effective than free drug in a mouse glioma xenograft model (94). The study demonstrated the general feasibility of the current approach for constructing targeted delivery systems based on the cell-penetrating peptides.

The BBTB is formed by brain tumor capillaries and comprises a barrier that is variably distinct from the BBB, forming an additional hurdle toward treatment. Lin et al. identified a novel BBB/BBTB-penetrating peptide M1 (TFYGGRPKRNNFLRGIR) from the phage displayed peptide library *in vivo* and modified the M1 peptide with a tumor-targeting named M1-RGD (TFYGGRPKRNNFLRGIRRGD), then they conjugated the MI-RGD with drug and applied PDC M1-RGD-PTX to treat glioma and found that it suppressed glioma proliferation and thus extended mouse survival in a glioma xenograft model (95). The study suggested that the peptide M1 could serve as a vector through the BBB and BBTB.

Therefore, the targeting peptides screening from the phage library are effective drug carriers across the BBB and BBTB, and phage display technology has wide applications for treating brain tumors.

## Ff Phage: A Potential Therapeutic Vehicle for Gliomas

The rod-shaped nanoparticles have higher avidity and selectivity for endothelial cells and increase the specificity and vascular targeting for brain endothelium (96). Increasing the length-to-diameter ratio of Ff phage results in more effective cellular attachment and ensuing membrane penetration. The phage maintains the biological activity of the peptide displayed on the phage vector, these properties make Ff phage suitable for use as a vector in the treatment of central nervous diseases (97), and research proved that phages carrying antibodies effectively label Aβ plaques is an efficient and nontoxic delivery vector to the brain and is useful for the treatment of Alzheimer’s disease *in vivo* (68).

### Ff Phage Could Deliver the Drug to the CNS

The function of BBB is under normal in low-grade glioma (98), Ff phage can pass the BBB and deliver therapeutics directly to the CNS when administered intranasally. It has been applied on protein-based treatments for other drug abuse syndromes. Janda et al. demonstrated that Ff phage displaying cocaine-binding proteins sequester cocaine in the brain and blocked the psychoactive effects of cocaine administered intranasally (23). Additionally, Ff phages have been reported to possess anti-tumorigenic properties. The researchers found that Ff phages could even inhibit the growth of subcutaneous GBM tumors in mice and this activity was mediated in part by lipopolysaccharide molecules attached to virion using the intranasal route.

Convection-enhanced delivery (CED) is a novel approach for administering chemotherapy in patients with brain tumors (99, 100). Additionally, CED is also an effective and safe method for distributing M13 phage to the brain (97). It reminds us that Ff phage could deliver the medicine to glioma *via* CED.

Normal vascular function is disturbed in high-grade glioma and Ff phage has more capacity to cross the BBB through various routes. Based on Ff phage, a functional dual vector could target and treat glioma intravenously. Hajitou et al. have designed a hybrid AAV/phage with a recombinant adeno-associated virus genome (rAAV) and the capsid of M13 phage as a vector for dual targeting of therapeutic genes to glioblastoma. The phage capsid displayed the RGD4C ligand that binds the αvβ3 integrin receptor and the recombinant rAAV genes expressed from a tumor-activated and temozolomide (TMZ)-induced promoter of the glucose-regulated protein, Grp78 (101). The recombinant vector targeted intracranial tumors in mice following intravenous administration and the gene delivered was expressed in human GBM cells. The construction of a double display Ff phage system was also reported. Sandlie and his team also developed a P III/P VII phage-genome double display system that could simultaneously carry two different exogenous peptides to perform different biological functions (102), and we can infer that the double display phage displayed targeting peptide and antibody could apply for treating glioma.

Taken together, Ff phages have the anti-tumor capability and could be genetically modified to display tumor homing motifs and conjugated to cytotoxic drugs. These phages are harmless when administered intranasally, CED, or intravenously and may present route anti-tumorigenic. Using them as vectors could be useful in the treatment of glioma.

### Future Prospects for Personalized Therapy

Glioma is a highly heterogeneous disease with major molecular differences in the expression of tumor cell surface markers in patients with the same grade of cancer (103). Currently, drugs used for glioma are often toxic to normal cells, resulting in serious side effects (104–106), and the broad range of drugs should be improved, as glioma cells are also prone to drug resistance (107, 108) Therefore, personalized therapy is very critical for gliomas.

Extensive research has used the phage display library to identify tumor-specific ligands by panning established tumor cell lines *in vitro* or by panning in an animal model. However, the material derived from the patient has more advantages of clinical relevance. It is tolerable in the human body, several groups have injected Ff phage library into patients without obvious side effects, and it is highly successful to develop a protocol for selecting phage displayed ligands in patients (109, 110). Shukla et al. conducted the toxicity profiles of different doses and phage displayed library formats for cancer patients (111). Then, they obtained and evaluated the tumor-homing phage-antibodies and derived soluble scFv antibodies to patients’ tumors and found that these antibodies were cancer-specific (112). Moreover, Ff phages are stable. They retain infectivity after IV injection and circulation in the human body. These studies remind us that phage display technology can be used to identify tumor-specific ligands to develop personalized therapy.

**Figure 2:**
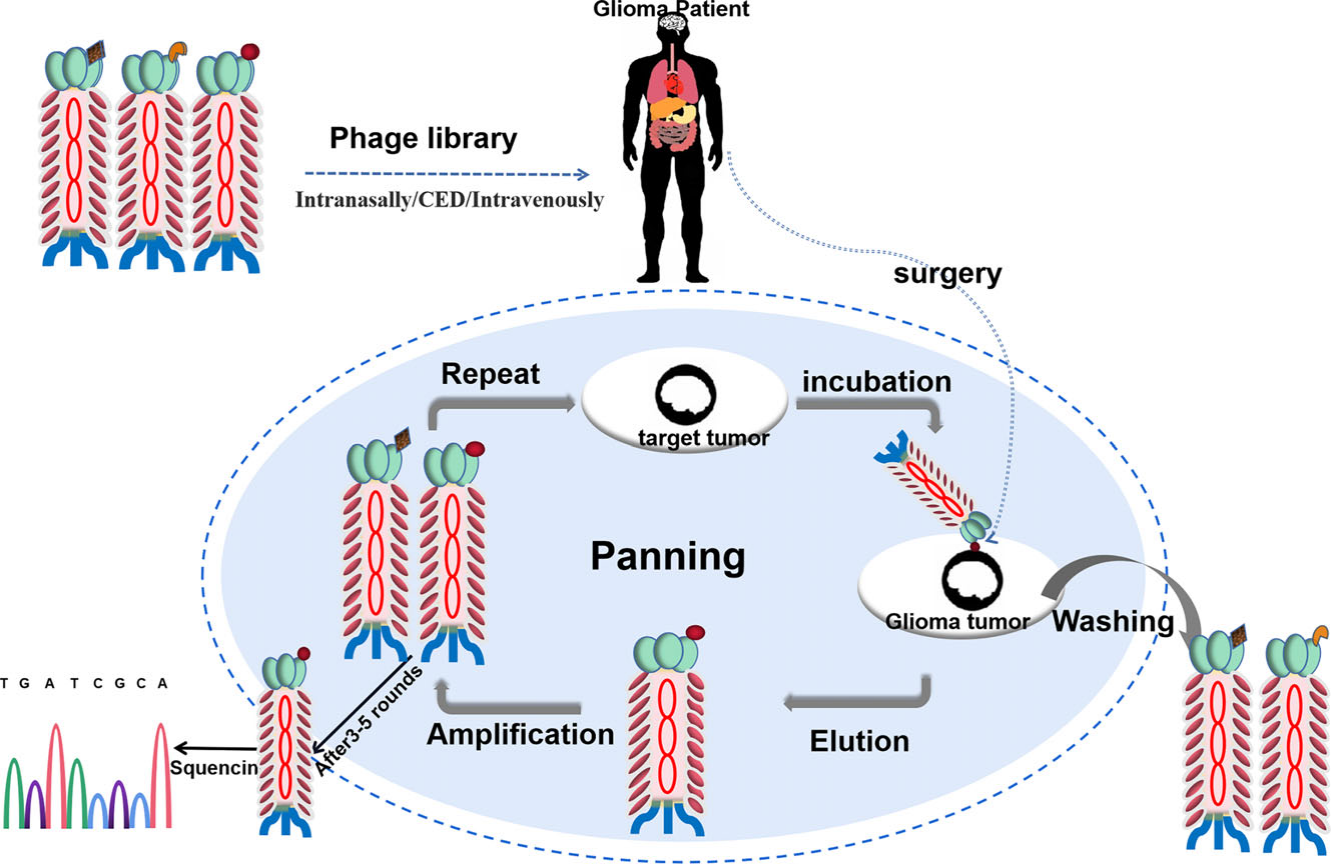
Schematic of affinity-selection of targets-binding phages from a phage library for personalized therapy of glioma. An antibody phage library is administered in glioma patients. After incubation, get the tumor from the patient. The unbound phages are washed and the bound phages are collected and amplified. After 3-5 rounds, the affinitive phages are enriched and sequenced.

Therefore, in theory, phage display strategies can achieve success when applied to target glioma cells for personalized treatments. Because of the specificity of biomarkers, glioma patients could be administered using phage display libraries and profiled for the presence of cancer targets before treatment. Such cancer-specific peptides can also be obtained from individual cancer patients *in vitro* and then be designed to target cancer treatment for personalized treatments (**Figure 2**).

## Conclusion

Gliomas are the most common primary brain tumors. Effective treatment of glioma is hampered by the presence of both BBB and BBTB. In this review, we presented an Ff phage approach to enhance the permeability of drugs through BBB and BBTB.

Although Ff phages have the problem of further optimization and improvement in separation and purification, they also have a number of advantages. Ff phage has a greater level of safety, it is not reproduce naturally in mammalian hosts, and it expresses a wide range of peptides on coat proteins using genetic engineering techniques to attach targeting peptides and antibodies.

Phage display is a high throughput screening strategy to construct peptide libraries that are used to screen glioma targeting peptides. These peptides might cross the BBB/BBTB and target tumors. It can also be used as a drug or drug carrier after being modified. Furthermore, Ff phage is an ideal transport carrier to CNS across the BBB, it has the anti-tumor ability and could be genetically modified to display glioma homing motifs and conjugated to cytotoxic drugs. Moreover, Ff phage displaying targeting peptide has stronger tumor penetrating ability, a higher load of drug delivery ability, and lower toxicity.

Developing carrier-based Ff phage as a drug delivery system can solve the problem of going through the BBB and BBTB. In short, Ff phage display technology is a powerful method of developing highly effective target drug delivery carriers. In addition, it opens the door to the development of personalized therapy agents in the future.

## Author Contributions

YW: Conceptualization; JS: Methodology and analysis; JC: Data Curation and analysis; CZ: Resources; XL: Visualization; WY: Writing; RC: Supervision; TG: Writing-Reviewing and Editing.

## Funding

This study was supported by the grant from the National Key R&D Program of China (Grant #2018YFC1311600), National Natural Science Foundation of China (81901880), Natural Science of Science and Technology Division Jilin (20200201505JC and 20190103099JH), Education deparment research project of Jilin (JJKH20201016KJ)and program of Jilin finance deparment (2019SRCJ003). Jilin provincial health project (2020SC2T091).

## Conflict of Interest

The authors declare that the research was conducted in the absence of any commercial or financial relationships that could be construed as a potential conflict of interest.

## Publisher’s Note

All claims expressed in this article are solely those of the authors and do not necessarily represent those of their affiliated organizations, or those of the publisher, the editors and the reviewers. Any product that may be evaluated in this article, or claim that may be made by its manufacturer, is not guaranteed or endorsed by the publisher.
